# **β**-Thalassemia Intermedia in Northern Iraq: A Single Center Experience

**DOI:** 10.1155/2014/262853

**Published:** 2014-02-27

**Authors:** Nasir A. S. Al-Allawi, Sana D. Jalal, Ameen M. Mohammad, Sharaza Q. Omer, Raji S. D. Markous

**Affiliations:** ^1^Scientific Research Center, Faculty of Medical Sciences, University of Duhok, Duhok 1014 AM, Iraq; ^2^Department of Pathology, Faculty of Medical Sciences, University of Sulaimaniyah, Sulaimaniyah, Iraq; ^3^Department of Internal Medicine, Faculty of Medical Sciences, University of Duhok, Duhok 1014 AM, Iraq; ^4^Department of Hematology, Shar Hospital, Sulaimaniyah, Iraq; ^5^Thalassemia Care Center, Duhok 1014 AM, Iraq

## Abstract

To investigate the molecular basis of **β**-thalassemia intermedia in Northern Iraq and evaluate its management practices, a total of 74 patients from 51 families were enrolled. The patients were clinically and hematologically reevaluated, and had their **β**-thalassemia mutations characterized, as well as the number of **α**-globin genes and *Xmn* I ^G^ **γ** −158 (C>T) polymorphism studied. Out of 14 **β**-thalassemia mutations identified, the four most common were IVS-I-6 (T>C) [33.3%], IVS-II-I (G>A) [21.1%], codon 82/83(−G) [10.1%], and codon 8 (−AA) [8.1%]. The most common contributing factors to the less severe phenotype of thalassemia intermedia were found to be the inheritance of mild **β**-thalassemia alleles and the *Xmn* I polymorphism, while concomitant **α**-thalassemia had a limited role. Several complications were documented including: pulmonary hypertension in 20.4%, diabetes mellitus in 1.4%, hypothyroidism in 2.9%, and heart failure in 2.7%, while no documented cases of venous thrombosis were found. Compared to their counterparts in several Mediterranean countries, it appears that our patients were much less frequently transfused and had a lower proportion of patients who were splenectomized, on iron chelation, or hydroxycarbamide therapy. Such practices require further scrutiny to ensure that a better level of care is provided and that growth retardation, skeletal changes, and other complications are prevented or reduced.

## 1. Introduction

Beta-thalassemia (thal) is an inherited autosomal recessive disorder due to reduction or absence of the hemoglobin *β*-globin chain synthesis, and it presents in one of three clinical phenotypes, namely, thalassemia major, minor, and intermedia. The former is due to homozygosity or compound heterozygosity to *β*-thalassemia mutations and is usually associated with lifelong dependence on blood transfusion and early presentation. Thalassemia minor, on the other hand, is classically an asymptomatic condition due to heterozygosity to a *β*-thalassemia defect [1]. Thalassemia intermedia (TI) is a less well-defined clinical entity which encompasses thalassemia patients with a wide spectrum of phenotypes that are more severe than thalassemia minor but milder than thalassemia major [2]. A variety of molecular mechanisms have been implicated, including the inheritance of mild *β*-thalassemia mutations, coinheritance of *α*-thalassemia, and inheritance of genetic determinants associated with high hemoglobin F production [3]. The contributions of these genetic modulators vary in different populations and their determination in these populations is imperative to tailor particular therapeutic strategies.

Several studies have addressed the molecular basis of thalassemia major and minor in Iraq [4–6], but none had looked at its basis in thalassemia intermedia patients. Therefore, the current study was initiated to determine the molecular basis of TI and the genotype/phenotype correlations in Northern Iraq and to review its management practices in comparison with those employed in other countries in the Mediterranean and the Middle East.

## 2. Patients and Methods

### 2.1. Patients

All patients diagnosed as thalassemia intermedia at the Duhok thalassemia care center in Northern Iraq were recalled. These constitute 20% of all registered *β*-thalassemia patients. A total of 79 patients responded and five were excluded because they did not fulfill the criteria for diagnosis of TI as set by the current study, leaving 74 patients from 51 families for inclusion. The criteria for diagnosis were based on those proposed by Qatanani and coworkers (2000), namely, first transfusion at/or after 2 years of age and/or absence of regular transfusion dependency. The distinction between thalassemia intermedia and minor was based on the presence of mild to severe anaemia with at least one of the following: (a) transfusion at some time during life; (b) splenomegaly or splenectomy; and (c) hemoglobin electrophoresis incompatible with thalassemia minor [7].

The study was approved by the ethical committee at the college of Medicine, University of Duhok, Duhok, Iraq and informed consent was obtained from all enrollees.

### 2.2. Clinical Evaluation

The enrolled patients had detailed clinical evaluation including a detailed history and physical examination. The patients' height and weight were checked to determine whether they had any growth retardation. The patients were scrutinized for skeletal face changes (thalassemic facies). The age at diagnosis and at first transfusion were recorded, as well as the number of transfusions over the last year. The spleen (unless splenectomized) and the liver sizes were checked. The records of all patients were checked for documented extramedullary hematopoiesis (EMH) as defined by radiological evidence of these extramedullary foci, regardless whether symptomatic or not. History of medications including iron chelation and hydroxycarbamide therapy was also sought.

### 2.3. Hematological Studies

At the time of enrolment all patients had their red cell indices determined using a hematology analyzer (Beckman Coulter, Fullerton, CA, USA) and Hb A_2_ and F by high performance liquid chromatography (VARIANT, Bio-Rad Laboratories, Hercules, CA, USA). The time of enrolment was just prior to the next transfusion, if the patient was on blood transfusion support. In patients who received blood at a rate of more than 4 occasions/year, the HPLC result at the time of diagnosis was quoted, if available.

### 2.4. DNA Studies

DNA was extracted from venous blood for all enrollees by a phenol-chloroform based method. The extracted DNAs were then screened for 20 *β*-thal mutations using multiplex polymerase chain reaction (PCR) and reverse hybridization, namely, −87 (C>G); −30 (T>A); codon 5 (−CT); codon 6 (−A); codon 8 (−AA); codons 8/9 (+G); codon 22 (−7 bp); codon 30 (G>C); IVS-I-1 (G>A); IVS-I-2 (T>A); IVS-I-5 (G>C); IVS-I-6 (T>C); IVS-I-110 (G>A); IVS-I-116 (T>G); IVS-I (−25 bp); codons 36/37 (−T); codon 39 (C>T); codon 44 (−C); IVS-II-1 (G>A); and IVS-II-745 (C>G). The amplification, hybridization, and detection procedures were performed as recommended by the manufacturer (Vienna Labordiagnostica GmbH, Vienna, Austria). For patients where the *β*-thal mutations remained uncharacterized by reverse hybridization, samples were sent for sequencing by Karminejad-Najmabadi Pathology and Genetic center in Tehran, Iran.

All patients were then screened for *Xmn *I ^G^ *γ* at −158 (C>T) polymorphism using a restriction fragment length polymorphism (RFLP) based method as detailed elsewhere [8]. Patient with the *Xmn *I ^G^ *γ* at −158 CC, CT, and TT genotypes were labeled as (−/−), (+/−), and (+/+), respectively. All patients were also screened for −*α* ^3.7^, −*α* ^4.2^, and the −−^MED−1^ *α*-thal deletions as well as the alpha triplication *ααα* by gap polymerase chain reaction, as described by Oron-Karni and coworkers [9].

### 2.5. Echocardiography

Echocardiography was offered to all patients at the time of the enrolment, after proper briefing on its clinical importance relevant to their management options; however, 54 patients chose to undergo it. The tricuspid regurgitant velocity (TRV) was calculated, and a TRV in excess of 2.8 m/s coupled with exertional dyspnea without evidence of left heart disease was considered as consistent with the diagnosis of pulmonary hypertension [10]. Heart failure was diagnosed based on the modified Framingham criteria [11].

### 2.6. Biochemical Studies and Other Tests

All patients had their serum ferritin assayed by an ELISA method (Biokit-USA), serum glucose (Biolabo-France), and serum alanine transaminase (ALT) (Biolabo-France) according to the manufacturer's instructions. The patients were also screened for Hepatitis B Surface antigen (HBsAg) (Plasmatic Laboratory Products-UK), Hepatitis C virus (HCV) antibodies (Plasmatic Laboratory Products-UK), and Human immune deficiency Virus (HIV) antibody 1 and 2 (Plasmatic Laboratory Products-UK). Serum thyroid stimulating hormone (TSH) and Free T4 were performed using enzyme immunoassay (TOSOH-Japan). Initially, patients were screened by TSH, if above 4.7 *μ*IU/mL, then Free T4 was performed, and if the latter is <0.8 ng/dL, then the patient was labeled as hypothyroid [12].

### 2.7. Statistical Analysis

Statistical analysis employed Mann Whitney *U* test and Chi Square (with or without Yates correction), when appropriate. A *P* < 0.05 was considered significant.

## 3. Results

### 3.1. Clinical Findings

Seventy-four patients (from 51 families) were enrolled in the current study. The patients had ages ranging between 2.5 years and 49 years (Median 15.5 yrs). They included 36 males and 38 females. The age of diagnosis varied between 3 months and 37 years (median 5 yrs). Thalassemic faces were noted in 54 cases (73%). Among the 51 patients who were 20 years or younger, 16 (31.3%) had heights less than the 3rd percentile and 14 (27.5%) had weights below 3rd percentile, while 12 had both height and weight below third percentile. None of the patients had ever had leg ulcers or documented venous thromboses. On reviewing patient's records, two patients had radiological evidence of EMH. One of the latter (patient number 71) had a hilar mass discovered on chest X-ray performed prior to splenectomy. The other patient (patient number 38) had a paraspinal EMH which led to spinal cord compression and paraplegia. The first patient was transfusion independent, while the second was off transfusion for about three years prior to enrolment.

### 3.2. Hematological Data

The patients had hemoglobins at the time of enrolment ranging between 6.5 and 11.6 g/dL (Mean ± SD = 8.25 ± 1.2) and MCVs ranging from 49 and 79 (Mean ± SD = 63 ± 7.3). Hemoglobin F prior to the next transfusions (or in those receiving >4 transfusions/year at the time of diagnosis) ranged between 3.3 and 98.5% (Mean ± SD =  54.1 ± 35%), while hemoglobin A_2_ ranged between 1.5 and 8.5% (Mean ± SD = 4.5 ± 2). Serum ferritin, on the other hand, ranged from 18 to 4853 *μ*g/L (Mean ± SD = 927.6 ± 999), including 24 (32.4%) having a ferritin in excess of 1000 *μ*g/L.  Table 1 outlines the main hematological parameters of all enrolled patients.

### 3.3. Molecular Studies

Table 2 summarizes the *β*-genotypes among the 51 families enrolled. A total of 14 *β*-thalassemia mutations were identified. The most frequent mutation encountered was IVS-I-6 (T>C) seen in 33 (33.3%), followed by IVS-II-1(G>A) in 21 (21.2%), codon 82/83 (−G) in 10 (10.1%), and codon 8 (−AA) in another 8 (8.1%). Other mutations were less frequent. The most frequent single genotype was IVS-I-6 (T>C)/IVS-I-6 (T>C), followed by IVS-II-1 (G>A)/IVS-II-1(G>A) and codon 82/83(−G)/codon 82/83(−G) (Table 2).

Fifteen families had homozygous or compound heterozygous *β* ^**+**^/*β* ^**+**^ mutations, while another 13 had *β* ^**+**^/*β* ^0^ genotypes. In 16 families, *β* ^0^/*β* ^0^ genotype were seen in association with *Xmn *I (+/+ or +/−) mutations, but no alpha deletions. In three other families, the *β* ^0^/*β* ^0^ was associated with *Xmn *I (+/−) and −*α* ^3.7^/*αα*, while in one family it was associated with neither. *Xmn* I polymorphism was most frequently encountered in association with IVS-II-I, codon 82/83, and codon 8. Three families had heterozygous *β*-thal, one of which had concomitant *ααα*/*αα* genotype (Table 2).

Except for the significantly older age at diagnosis (*P* = 0.033) and lower frequency of thalassemic faces (*P* = 0.036) in those with *β* ^+^/*β* ^+^ compared to those with *β* ^0^/*β* ^0^ genotypes, no other clinically significant findings were observed between the three main *β* genotype categories. On the other hand, Hb F levels were the least and Hb A_2_ the highest in those with *β* ^+^/*β* ^+^, which were significant when compared to either *β* ^0^/*β* ^0^ or *β* ^0^/*β* ^+^ genotypes, respectively (all with *P* < 0.0005). Moreover, *Xmn I* polymorphism was significantly less frequently encountered in families with *β* ^+^/*β* ^+^ genotype, when compared to either *β* ^0^/*β* ^+^ (*P* = 0.0001) or *β* ^0^/*β* ^0^genotype (*P* < 0.0000001).

### 3.4. Cardiac Studies

Tricuspid regurgitant velocity was in excess of 2.8 m/s in 11 out 54 patients (20.4%) who were screened for it. All patients with such an increase in TRV had variable degrees of exertional dyspnea and thus considered consistent with a diagnosis of pulmonary hypertension according to the criteria adopted by the current study [10]. Those with pulmonary hypertension were significantly older (*P* = 0.006), included more males (*P* = 0.042), were more likely to be splenectomized (*P* = 0.008) and had lower frequency of transfusions over their last year (1.77 ± 3.3 versus 1.0 ± 1.73, but this was not significant, *P* = 0.446), compared to those with no pulmonary hypertension. Heart failure was documented in two patients and one of whom died due to the failure, 3 months after enrolment.

### 3.5. Liver Function and Viral Screen

All patients were screened for Hepatitis B Surface Antigen, Hepatitis C virus, and HIV 1 and 2 by ELISA at the time of enrolment, and it was found that 1/74 (1.4%) was positive for HBsAg, while 13/74 (17.6%) were positive for HCV antibodies, and none were positive for HIV 1 and 2. Serum ALT was increased >50 IU/L at the time of enrolment in 10 patients (13.5%) including 6 of the HCV antibody and the only HBsAg positive cases.

### 3.6. Diabetes Mellitus and Hypothyroidism

A diagnosis of diabetes mellitus was made based on a fasting serum glucose >126 mg/dL on two occasions [14], in only one patient (1.4%). This patient was also suffering from heart failure and died 3 months after enrolment. On the other hand, of 70 patients who had their thyroid function evaluated, two had documented hypothyroidism (2.9%).

### 3.7. Management

Among the 74 enrolled patients, 24 patients (32.4%) were never transfused, while the remaining 50 patients had their first transfusions between ages of 1 and 42 years (median 5 years). The number of transfusion sessions that the patients had ranged between none and 12 over the last year prior enrolment. The majority had 3 or less transfusions annually (83.8%). Seventeen patients (23%) were splenectomized. Iron chelation therapy of at least one year duration at the time of enrolment, included deferoxamine (Desferal) in eight patients and deferasirox (Exjade) in three patients. Seven other patients were recently initiated on oral iron chelation with deferasirox as it became freely available from health authorities. Only two patients were on hydroxycarbamide at the time of enrolment and both were transfusion free over the last year.

## 4. Discussion

### 4.1. The molecular Basis of *β*-Thalassemia Intermedia

The most frequent contributor to *β*-thalassemia intermedia in Northern Iraq was found to be the inheritance of *β* ^+^ mutations (*β* ^+^/*β* ^+^ or *β* ^+^/*β* ^0^), and this was seen in 28/51 families (54.9%). This is to a considerable extent comparable with studies from some Mediterranean countries like Lebanon and Italy, where the inheritance of mild *β* ^+^ alleles is responsible for the majority of their TI cases [7, 15]. Another frequently encountered contributor was* Xmn* I polymorphism which was seen in 19/20 families (95%) with *β* ^*ο*^/*β* ^*ο*^ and in 9/13 (69.2%) with *β* ^*ο*^/*β* ^+^ but in none (0%) of those with *β* ^+^/*β* ^+^ or those with *β* ^*ο*^/wild genotypes (Table 2). This is consistent with the findings of Verma and coworkers (2007) who demonstrated a similar pattern among 325 TI patients from different ethnic backgrounds in the Mediterranean region and Asia and concluded that this polymorphism is the commonest ameliorating factor in cases with *β* ^0^ mutations but not *β* ^+^ [16]. Accordingly in neighboring Iran, where *β* ^0^ mutations are more frequent than *β* ^+^ in TI patients, *Xmn* I polymorphism was reported as the most frequent ameliorating factor [17], while the latter polymorphism comes second in importance in the Lebanese and the Italians [7, 15], and it is almost irrelevant among Cypriots TI where *β* ^+^ mutations predominate [16]. The* Xmn* I polymorphism is one of three major Hb F quantitative trait loci (QTLs) responsible for Hb F variation, and it leads to a less severe phenotype by increasing *γ* chain production, which helps to neutralize unbound *α*-chains [18, 19].

Concomitant alpha-thalassemia in association with homozygous or compound heterozygous *β*-thal would reduce the excess alpha chains and thus lead to a less severe phenotype [1]. However, it was not found to be an important contributor to TI phenotype in Iraqi patients, and was seen in four patients only, all of whom were also heterozygous for the *Xmn* I polymorphism. The relative lack of contribution of *α*-thal is shared by similar studies from Iran and Lebanon [7, 17] but is in contrast to others from Cyprus and India [16, 20], where *α*-thal deletions are seen in 35.3% and 38% of TI patients, respectively.

In only three out of the 51 families (5.9%), the patients had heterozygous *β*-thalassemia, and this is consistent with previous studies worldwide where the large majority of cases of TI was homozygous or compound heterozygous to *β*-thalassemia mutations [19]. In one of these heterozygous patients there was a concomitant *ααα*/*αα* genotype, which would explain the TI phenotype in these patients and such alpha triplication increases the *α* : *β* imbalance among *β*-thal heterozygotes and has been implicated in TI in several earlier studies from Asia and the Mediterranean region [13, 15, 16, 19–21]. The other two heterozygous cases were associated with a normal alpha genotype, and thus their TI remains unexplained and we postulate that further unidentified genetic and/or environmental factors may be implicated.

Another unexplained case was case number 70, where a *β* ^0^/*β* ^0^ (IVS-I-130/Codon 5), was associated with (−/−) *Xmn* I status and no concomitant *α*-thal deletions. The latter patient may have nondeletional *α*-thal, which was not part of the investigations in the current study or may have other molecular mechanisms that require further scrutiny.

The most frequent mutations vary in different geographical locations, and in the current study the most common was IVS-I-6, a mild *β* ^+^ mutation, which is consistent with that seen in several Mediterranean countries like Cyprus, Italy, and Lebanon [7, 15, 16, 19]. IVS-II-1 was the second most common mutation encountered in our patients, while it was the commonest one among Iranian TI patients [17]. In both our study and the latter Iranian study, the milder phenotype was linked with *Xmn* I polymorphism. In other Asian countries, the most frequent mutations varied from IVS-I-1 (G>T) in India, IVS-I-5 (G>C) in Pakistan [16], to −28 (A>G) in Southern China [13]. The current study showed that codon 82/83, a *β* ^*ο*^ mutation associated with *Xmn* I polymorphism, was the third most common mutation, and the current report is the first to report this mutation from Iraq. This mutation was also reported in association with thal intermedia in Iran [17].

### 4.2. Current Management Practice

The practices in TI management employed in several Mediterranean and Middle Eastern countries have been reviewed recently by the Optimal Care study [22], and when we compared our results with those of the latter overview, we found that our patients were rather younger, were much less regularly transfused, and included much lower proportions of patients who were splenectomized, chelated, or on fetal hemoglobin modulating therapies (Table 3). This state is clearly reflective of the current practice of limited intervention adopted by the physicians caring for TI in our center, particularly with minimal use of blood transfusions. However, such a practice may have contributed to the higher degrees of growth retardation and skeletal changes in the current study compared to its counterparts in some Mediterranean countries, like Lebanon [7], though it is better than those in some Indian and Iranian centers where transfusions were even more sparingly used than in our study [23, 24]. It is important to note that the lower rate of transfusions may also lead to higher rate of complications, and thus a lower health-related quality of life [HRQoL], as shown by a recent study by Musallam and coworkers [25]. Thus it is important to acknowledge the need to review the transfusion practices and follow preset guidelines as outlined by several recent reviews, a process which is currently underway [26].

Despite the less regular transfusions, about a third of our patients had serum ferritins in excess of 1000 ug/L, which is to a great extent comparable to that reported by the Optimal Care study (Table 3) [22]. This is mainly related to the fact that they were less regularly chelated compared to the former study. Iron overload is a recognized complication of TI [27]. The cause of iron overload in TI is likely to be due to a combination of ineffective erythropoiesis, anemia, and hypoxia leading to a compensatory increase in erythropoietin and a decrease in serum hepcidin. The latter will trigger increased intestinal iron absorption with preferential liver iron accumulation [2]. The use of serum ferritin to monitor iron overload is inexpensive and accessible; however, in TI serum ferritin assays may underestimate the actual iron load and liver iron concentration (LIC) is more reliable, though invasive [27]. Magnetic resonance imaging (MRI) has been advocated as an alternative noninvasive reliable procedure for assessing LIC [27]. However and as with many developing countries, neither LIC nor MRI are available to our patients. The threshold ferritin level for considering initiating chelation therapy is lower than that accepted for thalassemia major. Taher and coworkers (2013) proposed an algorithm for treating iron overload in Nontransusion Depenedent Thalassemia (including TI), where a cut-off serum ferritin for initiating chelating therapy was set at 800 *μ*g/L. Applying the latter algorithm would have led to chelating more of our patients. The main obstacle was, until recently, the high rates of noncompliance with relatively cumbersome deferoxamine therapy. However, the introduction of the oral iron chelator deferasirox, and its reported effectiveness in thalassemia intermedia as demonstrated by several studies [28, 29], may offer a viable option for our patients, as it is now provided freely by the local health authorities.

One complication which was previously overlooked but was focused upon in this cross-sectional study was pulmonary hypertension, and interestingly it was found to be nearly double that reported by the Optimal Care study of 11% [22], despite the fact that the current study included younger and less splenectomized patients, and this may again be due to the policy of minimal use of transfusion therapy. Pulmonary hypertension in TI is multifactorial with several possible mechanisms, one of which is related to the chronic hemolytic process leading to a high output state and resultant vascular changes, another mechanism is nitrous oxide depletion, leading to endothelial dysfunction and pulmonary vasculature remodeling [30]. Thus our patients who had higher degrees of anemia would be at a higher risk of pulmonary hypertension. The association of pulmonary hypertension with older age and splenectomy as documented by the current study is consistent with previous studies on TI [22, 31, 32]. It is important to note that similar to several previous studies on pulmonary hypertension in TI, we used the TRV as a diagnostic tool, and it is well known that this approach is less reliable than catheterization to determine the pulmonary artery pressure directly; however, by using a higher cut-off point of 2.8 m/s (instead of the 2.5 m/s used by some previous investigators), coupled with exertional dyspnea, we aimed at reducing false positive results [10, 22, 30]. A recent Italian report proposed adopting an even higher TRV cut-off point of 3.2 m/s, as a more specific determinant of pulmonary hypertension. In the current study six patients (11.1%) had TRV ≥ 3.2 m/s, which is still nearly double that reported by the Italian workers in their TI patients [32].

Other complications including heart failure, hypothyroidism and diabetes mellitus were documented in 2.7%, 2.9%, and 1.4%, respectively, which is more or less comparable to rates reported in surrounding countries [22, 24]. It is interesting, however, to note that no cases of thrombosis were documented by the current study in contrary to several studies from the region [22, 33]. The latter may be related to the possibility that some cases may have been asymptomatic, but more likely that this age and splenectomy related complication is less likely in our younger and less splenectomized patients compared to the Optimal Care study (Table 3) [22, 34]. Despite the fact that many of the important complication of TI were evaluated in this cross-sectional study, few important complications like osteoporosis and hypogonadism, were not formally evaluated at the time of enrolment, which is a drawback of the current study and should be included in future studies.

Finally it is worth noting that liver enzymes were abnormal in 13.6%, with the majority associated with HCV antibodies. The latter were seen in 17.6%, which is intermediate between the figures quoted from Lebanon (7%) and Italy (33%), though they are much less than the rate of 56.9% in thalassemia major in our center (unpublished observations) as anticipated [35].

Hydroxycarbamide, a therapeutic modality which has shown benefit in TI by several studies through fetal hemoglobin induction [36], was only sparingly used by the physicians at our center, and its use was restricted to two patients who refused further transfusions following transfusion reactions. This is in contrast to its much wider use in several centers in Middle East [22] as documented by the Optimal Care study.

## 5. Conclusions

The current study, which is the first from Iraq on genotype/phenotype correlations in thalassemia intermedia, revealed that the inheritance of milder *β*-thalassemia mutations and the *Xmn* I polymorphism are the two most important mechanisms implicated, while concomitant *α*-thal mutations had evidently a limited role. It appears also that the limited intervention adopted especially with blood transfusion may have lead to higher proportion of patients with skeletal and/or growth retardation. It was also noted that a number of patients were denied the benefits of chelation therapy and/or hydroxyurea, mainly because of their families or their own personal choices. The current study highlighted the need for patients to be better informed of the natural history of their disease, its long term complications and available therapy options that may prevent such complications, and also to seriously reconsider the transfusion and chelation policies employed with the ultimate aim of providing the best possible care.

## Figures and Tables

**Table 1:** Genotype, clinical, and hematological parameters in 74 patients with TI enrolled in the current study.

Number	Family	*β*-Genotype	*Xmn *I status	*α*-Genotype	Age (yr)	Sex	Age at diagnosis (yr)	Age at first Tx.	Number of Tx. last yr.	Spleen cm BCM	Hb (g/dL)	A_2_ (%)	*F* (%)
1	F1	IVS-I-6/IVS-I-6	−/−	*αα*/*αα*	20	F	8	8	0	5	7.5	7.1	8.7
2	F1	IVS-I-6/IVS-I-6	−/−	*αα*/*αα*	21	M	10	10	0	**S**	8.0	8.4	11.1
3	F2	IVS-I-6/IVS-I-6	−/−	*αα*/*αα*	7	M	4	NT	0	3	7.0	6.9	8.7
4	F2	IVS-I-6/IVS-I-6	−/−	*αα*/*αα*	9	F	7	NT	0	3	7.6	6.1	14.9
5	F3	IVS-I-6/IVS-I-6	−/−	*αα*/*αα*	13	M	5	12	1	3	8.1	6.6	24.5
6	F4	IVS-I-6/IVS-I-6	−/−	*αα*/*αα*	19	F	4	4	2	3	7.5	5.8	17.6
7	F4	IVS-I-6/IVS-I-6	−/−	*αα*/*αα*	21	M	4	4	1	4	9.1	7.0	8.3
8	F5	IVS-I-6/IVS-I-6	−/−	*αα*/*αα*	38	F	24	24	4	**S**	9.3	8.0	10.0
9	F6	IVS-I-6/IVS-I-6	−/−	*αα*/*αα*	27	F	4	26	0	5	7.8	6.8	10.4
10	F6	IVS-I-6/IVS-I-6	−/−	*αα*/*αα*	33	M	5	5	0	**S**	8.2	8.5	7.7
11	F7	IVS-I-6/IVS-I-6	−/−	*αα*/*αα*	10	F	8	NT	0	1	7.3	7.2	9.8
12	F7	IVS-I-6/IVS-I-6	−/−	*αα*/*αα*	11	F	10	NT	0	1	7.3	6.4	12.3
13	F7	IVS-I-6/IVS-I-6	−/−	*αα*/*αα*	7	F	5	NT	0	NP	7.4	6.2	13.0
14	F8	IVS-I-6/IVS-I-6	−/−	*αα*/*αα*	5	M	1	1	2	3	7.8	4.5	17.3
15	F9	IVS-I-6/IVS-I-6	−/−	*αα*/*αα*	4	M	3 m	NT	0	2	7.6	4.8	36.3
16	F10	IVS-I-6/IVS-I-6	−/−	*αα*/*αα*	3.0	M	3	NT	0	2	10.6	4.2	32.5
17	F11	IVS-I-6/IVS-I-6	−/−	*αα*/*αα*	31	F	6	6	12	3	9.1	NA	NA
18	F12	IVS-I-6/IVS-I-6	−/−	*αα*/*αα*	7	F	5	NT	0	7	7.2	7.3	29.4
19	F12	IVS-I-6/IVS-I-6	−/−	*αα*/*αα*	15	M	5	5	0	7	6.8	7	26.9
20	F13	IVS-I-128/IVS-I-128	−/−	*αα*/*αα*	24	M	11	NT	0	1	8.6	7	25
21	F13	IVS-I-128/IVS-I-128	−/−	*αα*/*αα*	21	F	5	5	3	1	7.9	5	35
22	F14	−28/−28	−/−	*αα*/*αα*	33	M	15	15	2	6	7.6	7	18.5
23	F15	IVS-1-6/−30 (T>A)	−/−	*αα*/*αα*	33	F	11	11	0	**S**	8.3	5	20
24	F16	IVS-I-6/IVS-II-1	+/−	*αα*/*αα*	8	M	7.5	NT	0	4	9.2	3.9	65.7
25	F17	IVS-I-6/IVS-II-1	+/−	*αα*/*αα*	10	M	10	NT	0	2	8.3	3	62.2
26	F18	IVS-I-6/Cd 82/83	+/−	*αα*/*αα*	6	F	9 m	5	1	2	7.7	2.8	54.2
27	F18	IVS-I-6/Cd 82/83	+/−	*αα*/*αα*	4	M	9 m	NT	0	1	8.1	1.5	45.5
28	F18	IVS-I-6/Cd 82/83	+/−	*αα*/*αα*	3	F	3	NT	0	2	8.3	2.0	60
29	F18	IVS-I-6/Cd 82/83	+/−	*αα*/*αα*	13	F	6	6	10	1	8.9	2.8*	45*
30	F19	IVS-I-6/Cd 39	−/−	*αα*/*αα*	5	F	6 m	2	1	3	8.0	3.5	56.0
31	F20	IVS-I-6/Cd 5	−/−	*αα*/*αα*	46	F	34	40	0	**S**	7.8	5.1	87.2
32	F20	IVS-I-6/Cd 5	−/−	*αα*/*αα*	49	F	37	42	0	**S**	7.2	8	80.1
33	F21	IVS-1-6/Cd 8	+/−	*αα*/*αα*	25	F	24	24	8	7	8.0	3.3*	58*
34	F22	IVS-I-6/Cd 8	+/−	*αα*/*αα*	2.5	F	2.5	2.5	1	2	6.9	5.5	46.6
35	F22	IVS-I-6/Cd 8	+/−	*αα*/*αα*	13	F	5	5	12	**S**	9.6	NA	NA
36	F22	IVS-I-6/Cd 8	+/−	*αα*/*αα*	5	F	4	4	2	4	7.2	6.1	48
37	F23	IVS-1-6/IVS-I-1	−/−	*αα*/*αα*	29	F	6	6	12	**S**	10.6	NA	NA
38	F24	IVS-I-130/Poly A	−/−	*αα*/*αα*	32	M	8	8	0	7	6.5	3	46
39	F25	Cd 5/Poly A	+/−	*αα*/*αα*	16	M	3	3	3	7	7	4.2	35.4
40	F25	Cd 5/Poly A	+/−	*αα*/*αα*	18	M	3	3	0	**S**	6.7	5.7	37.9
41	F25	Cd 5/Poly A	+/−	*αα*/*αα*	7	F	3	3	4	4	6.8	4.0	33.9
42	F25	Cd 5/Poly A	+/−	*αα*/*αα*	22	F	2	2	0	**S**	7.9	6.4	35.4
43	F25	Cd 5/Poly A	+/−	*αα*/*αα*	8	F	6	NT	0	2	7.1	3.8	49.1
44	F26	Cd 5/Poly A	+/−	*αα*/*αα*	23	F	5	5	0	4	8.6	6.1	41.6
45	F26	Cd 5/Poly A	+/−	*αα*/*αα*	36	F	17	17	0	3	7.3	6.6	37.1
46	F26	Cd 5/Poly A	+/−	*αα*/*αα*	19	M	7	7	0	3	7.4	6.3	36.8
47	F27	IVS-II-1/IVS-II-1	+/+	*αα*/*αα*	19	M	1	NT	0	**S**	9.5	1.5	98.5
48	F27	IVS-II-1/IVS-II-1	+/+	*αα*/*αα*	8	M	1	NT	0	**S**	9.3	3	96
49	F28	IVS-II-1/IVS-II-1	+/+	*αα*/*αα*	14	M	10	NT	0	4	11	3.5	96.5
50	F28	IVS-II-1/IVS-II-1	+/+	*αα*/*αα*	17	F	3	3	1	5	11.6	4.0	96
51	F29	IVS-II-1/IVS-II-1	+/+	*αα*/*αα*	7	F	4	4	0	6	7.5	1.8	96.3
52	F30	IVS-II-1/IVS-II-1	+/+	*αα*/*αα*	18	M	16	NT	0	2	10	2.6	97
53	F31	Cd 82/83/Cd 82/83	+/+	*αα*/*αα*	29	M	9	9	4	**S**	9.9	2.7	91.7
54	F32	Cd 82/83/Cd 82/83	+/+	*αα*/*αα*	26	M	5	5	0	10	7.6	2.5	97.5
55	F33	Cd 82/83/Cd 82/83	+/+	*αα*/*αα*	25	M	19	19	0	1	11.0	1.6	98.3
56	F34	Cd 82/83/Cd 82/83	+/+	*αα*/*αα*	8	M	2.5	2.5	0	4	7.3	3.8	95
57	F35	Cd 8/Cd 8	+/+	*αα*/*αα*	19	F	7	7	3	**S**	9.4	2.0	94
58	F36	IVS-II-1/Cd 82/83	+/+	*αα*/*αα*	9	M	1	1	5	3	9.5	4.7*	95.0*
59	F37	IVS-II-1/Cd 8	+/+	*αα*/*αα*	7	M	7	NT	0	3	7.5	2	98
60	F38	Cd 8/IVS-II-1	+/−	*αα*/*αα*	12	M	5	5	12	2	6.9	2.5*	97.5*
61	F38	Cd 8/IVS-II-1	+/−	*αα*/*αα*	5	F	9 m	NT	0	1	8.7	2.0	98
62	F39	IVS-II-1/Cd 44	+/−	*αα*/*αα*	5	F	2	2.5	2	2	8.1	1.8	98.2
63	F40	IVS-II-1/Cd 44	+/−	−*α* ^3.7^/*αα*	18	M	2	NT	0	2	9.7	2.3	97.7
64	F41	IVS-II-1/IVS-I-1	+/−	*αα*/*αα*	3	M	8 m	NT	0	NP	7.4	3	96.8
65	F42	IVS-II-1/IVS-I-1	+/−	−*α* ^3.7^/*αα*	5	M	4	4	10	1	6.7	2*	98*
66	F43	Cd 8/IVS-I-110	+/−	−*α* ^3.7^/*αα*	21	M	1	1	0	**S**	10	5	87
67	F44	IVS-II-1/IVS1-110	+/−	*αα*/*αα*	12	M	5	5	2	7	8.3	2.9	55
68	F45	Cd 8/IVS-I-1	+/−	*αα*/*αα*	4	M	2	4	1	1	7.7	1.6	98
69	F46	IVS-II-1/IVS-II-1	+/−	−*α* ^3.7^/*αα*	18	F	6	6	0	3	10	2.7	97.3
70	F47	IVS-I-130/Cd 5	−/−	*αα*/*αα*	16	F	2	2	0	**S**	9.6	2.5	97.4
71	F48	IVS-II-1/IVS-1-130	+/−	*αα*/*αα*	20	F	3	NT	0	**S**	8.3	5	95
72	F49	Cd 5/Wild	−/−	*αα*/*αα*	8	F	2	NT	0	3	6.9	5.8	5.7
73	F50	Cd 5/Wild	−/−	*αα*/*ααα*	17	F	13	13	4	5	6.5	4.5	3.3
74	F51	Cd 39/Wild	−/−	*αα*/*αα*	30	M	19	19	1	1	9	4.9	8.8

NA: Not Available; NP: Not Palpable; *Parameter at diagnosis; S: Splenectomy; NT: never transfused; M: Male; F: Female; Poly A: AATAAA>AATAAG mutation; Tx: transfusion.

**Table 2:** The *β*-and *α*-genotypes and *Xmn *I polymorphism status in the 51 families enrolled in the current study.

*β*-Genotype	*Xmn *I	Alpha genotype	Number
*β* ^+^/*β* ^+^			
IVS-1-6 (T>C)/IVS-I-6 (T>C)	−/−	*αα*/*αα*	12
IVS-I-128 (T>G)/IVS-I-128 (T>G)	−/−	*αα*/*αα*	1
−28 (A>C)/−28 (A>C)	−/−	*αα*/*αα*	1
IVS-I-6 (T>C)/−30 (T>A)	−/−	*αα*/*αα*	1
*β* ^+^/*β* ^0^			
IVS-I-6 (T>C)/IVS-II-1 (G>A)	+/−	*αα*/*αα*	2
IVS-I-6 (T>C)/Codon 8 (−AA)	+/−	*αα*/*αα*	2
Poly A (AATAA*A*>AATAA*G*)/Codon 5 (−CT)	+/−	*αα*/*αα*	2
IVS-I-6 (T>C)/Codon 5 (−CT)	−/−	*αα*/*αα*	1
IVS-I-6 (T>C)/Codon 39 (C>T)	−/−	*αα*/*αα*	1
IVS-I-6 (T>C)/Codon 82/83 (−G)	+/−	*αα*/*αα*	1
IVS-I-6 (T>C)/IVS-I-1 (G>A)	−/−	*αα*/*αα*	1
Poly A (AATAA*A*>AATAA*G*)/IVS-1-130 (G>C)	−/−	*αα*/*αα*	1
IVS-II-1 (G>A)/IVS-I-110 (G>A)	+/−	*αα*/*αα*	1
Codon 8 (−AA)/IVS-I-110 (G>A)	+/−	−*α* ^3.7^/*αα*	1
*β* ^0^/*β* ^0^			
IVS-II-I (G>A)/IVS-II-1 (G>A)	+/+	*αα*/*αα*	4
Codon 82/83 (−G)/Codon 82/83 (−G)	+/+	*αα*/*αα*	4
Codon 82/83 (−G)/IVS-II-1 (G>A)	+/+	*αα*/*αα*	1
Codon 8 (−AA)/Codon 8 (−AA)	+/+	*αα*/*αα*	1
Codon 8 (−AA)/IVS-II-1 (G>A)	+/+	*αα*/*αα*	1
Codon 8 (−AA)/IVS-II-1 (G>A)	+/−	*αα*/*αα*	1
Codon 8 (−AA)/IVS-I-1 (G>A)	+/−	*αα*/*αα*	1
IVS-II-1 (G>A)/Codon 44 (−C)	+/−	*αα*/*αα*	1
IVS-II-1 (G>A)/IVS-I-1 (G>A)	+/−	*αα*/*αα*	1
IVS-II-1 (G>A)/IVS-I-130 (G>C)	+/−	*αα*/*αα*	1
IVS-II-1 (G>A)/IVS-II-1 (G>A)	+/−	−*α* ^3.7^/*αα*	1
IVS-II-1 (G>A)/Codon 44 (−C)	+/−	−*α* ^3.7^/*αα*	1
IVS-II-1 (G>A)/IVS-I-1 (G>A)	+/−	−*α* ^3.7^/*αα*	1
IVS-I-130 (G>C)/Codon 5 (−CT)	−/−	*αα*/*αα*	1
*β* ^0^ */wild *			
Codon 5 (−CT)/wild	−/−	*αα* *α*/*αα*	1
Codon 5 (−CT)/wild	−/−	*αα*/*αα*	1
Codon 39 (C>T)/wild	−/−	*αα*/*αα*	1

**Table 3:** A comparison between some parameters and treatment options in the current study and the optimal care study [22].

Parameter	Optimal care study	Current study
Age (yr)		
<18	29.5	55.4
18–35	49.3	39.2
>35	21.2	5.4
Male : female	1 : 1	1 : 1.1
Serum ferritin *μ*g/L		
<1000	64.4	67.6
1000–2500	30.6	27.0
>2500	5	5.4
Treatment		
Hydroxyurea	34.6	2.7
Occasional transfusion (0–3/yr)	24.5	83.8
Regular transfusion (>3/y)	51.7	16.2
Iron chelation (>1 year)	47.5	14.9
Splenectomized	55.7	23.0
